# Relative Grip Strength as a Screening Indicator for Metabolic Syndrome in Korean Adults: A Cross-Sectional Study Using Data from KNHANES

**DOI:** 10.3390/medicina61081473

**Published:** 2025-08-16

**Authors:** Jongsuk Park, Sangho Kim

**Affiliations:** 1 Department of Sport and Health Studies, Konkuk University, Chungju-si 27478, Republic of Korea; model200@kku.ac.kr; 2 School of Global Sport Studies, Korea University, Sejong-si 30019, Republic of Korea

**Keywords:** grip strength index, metabolic screening, lifestyle-related disease, public health monitoring, muscle assessment, population-based study

## Abstract

*Background and Objectives*: This study aimed to assess the association between relative grip strength (RGS) and metabolic syndrome (MetS) in Korean adults and to explore sex- and age-specific cutoff values for screening purposes. *Materials and Methods*: This cross-sectional study analyzed data from 12,072 Korean adults (aged 19–64 years) who participated in the 2017–2019 Korea National Health and Nutrition Examination Survey. Receiver operating characteristic (ROC) curve analysis was performed stratified by sex and age group, and participants were categorized into adequate (ARG) and low (LRG) RGS groups. Multivariable logistic regression was used to examine the association between RGS (as both categorical and continuous variables) and MetS, adjusting for lifestyle and behavioral covariates. *Results*: The RGS demonstrated moderate discriminatory power for MetS, with area under the curve (AUC) values ranging from 0.601 to 0.742. Age- and sex-specific cutoff values for RGS progressively decreased with age. Individuals in the LRG group had significantly higher odds of MetS across all age and sex groups. The LRG group had significantly greater odds of MetS in nearly all subgroups (e.g., women aged 20–39 years: odds ratio [OR] = 6.846; men: OR = 3.502). As a continuous variable, each 0.1-unit increase in RGS was associated with a 22.1–33.4% reduction in the odds of MetS (*p* < 0.001). *Conclusions*: RGS is inversely associated with MetS, particularly in women and younger adults. Although its discriminatory ability is moderate, the RGS may serve as a simple and accessible screening indicator to help identify individuals with an increased metabolic risk.

## 1. Introduction

Metabolic syndrome (MetS) is a complex pathological condition characterized by a cluster of interrelated risk factors, including abdominal obesity, hypertension, insulin resistance, and dyslipidemia, which collectively increase the risk of cardiovascular disease and type 2 diabetes [1]. Globally, the prevalence of MetS is estimated to range from 12.5% to 31.4% [2], with a consistent upward trend observed in recent decades. In South Korea, the prevalence of MetS has increased from 27.0% in 2007 to 33.2% in 2020 [3], highlighting its growing importance in public health. Given its strong association with cardiovascular and cerebrovascular diseases and its contribution to elevated mortality rates, early identification of at-risk individuals is essential for timely intervention and effective disease prevention.

Grip strength (GS) is an inexpensive, non-invasive, and easily measurable clinical indicator of overall muscular strength. It is a valuable predictor of general health and the risk of chronic diseases [4]. Several studies have reported that a lower GS is significantly associated with an increased risk of chronic diseases and multimorbidity [5,6]. Additionally, several large-scale epidemiological studies have identified an inverse relationship between GS and the prevalence of MetS and its components [6,7,8]. Mechanistically, GS reflects both muscle strength and physical activity levels, which are crucial for maintaining glucose metabolism and insulin sensitivity [9]. As skeletal muscle is the primary site of glucose uptake, its functional decline can significantly contribute to the pathogenesis of MetS [10,11,12]. Muscle strength, which reflects an individual’s physical activity level and muscle mass, is frequently used as a predictive marker for the risk of metabolic disease [13]. Therefore, GS could serve as a valuable clinical indicator for assessing the risk of MetS and underscores the importance of maintaining muscle strength to mitigate this risk [14].

Although GS has shown clinical promise, its interpretation is confounded by body size. Absolute GS values are often skewed by variations in weight or body mass index (BMI), limiting their diagnostic value in diverse populations [15]. The same absolute GS level may have different metabolic implications depending on an individual’s weight or BMI [16,17]. To overcome this limitation, relative GS (RGS), defined as absolute GS divided by BMI or weight, has been proposed as a more robust and size-adjusted indicator of metabolic health [4,16]. Prior research suggests that RGS is more consistently associated with metabolic health outcomes than absolute GS [15,18]. Importantly, a lower RGS is associated with higher odds of prevalence across all age groups, whereas absolute GS exhibits inconsistent associations [19].

Despite increasing interest in RGS as a functional biomarker, few studies have systematically evaluated its diagnostic performance using receiver operating characteristic (ROC) analysis, particularly in Asian populations. To date, no study has established optimal RGS cutoff values for predicting MetS in Korean adults based on population-level data. The lack of validated thresholds constrains the clinical application of the RGS as a screening tool, facilitating early intervention and personalized health management strategies. Given its simplicity, cost-effectiveness, and non-invasive nature, incorporating RGS measurements into routine health evaluations could reduce the prevalence of MetS, improve the quality of life, and alleviate the economic burden of chronic disease management.

This study addresses this critical gap by using nationally representative data from the Korea National Health and Nutrition Examination Survey (KNHANES) to determine sex-specific RGS cutoff values predictive of MetS using ROC analysis. By providing clinically actionable thresholds, this study provides novel evidence for integrating the RGS into routine metabolic health screening. Furthermore, it expands upon previous work by validating the RGS in a large, ethnically homogeneous cohort, reinforcing its potential as a scalable and cost-effective marker for the early detection and prevention of MetS in Korean adults.

## 2. Materials and Methods

### 2.1. Study Population

This study employed a cross-sectional design using raw data from the KNHANES, a nationally representative and reliable dataset designed to evaluate health behaviors, health status, and dietary and nutritional intakes within the Korean population. GS measurements required for this study were collected as part of the KNHANES from 2014 to 2019. The most recent raw datasets from 2017 to 2019 were used for this analysis.

**Figure 1:**
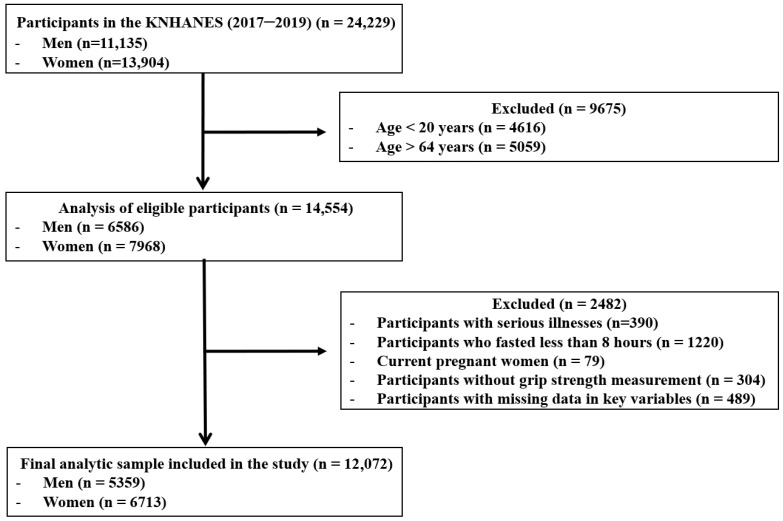
Flow diagram of inclusion and exclusion of study participants. KNHANES: Korean National Health and Nutrition Examination Survey.

The initial survey population consisted of 24,229 individuals who participated in the KNHANES between 2017 and 2019. The following exclusion criteria were applied: participants outside the specified age range of 19–64 years (*n* = 9675); participants with a self-reported history of cardiovascular disease, stroke, or cancer (*n* = 390); individuals who did not adhere to an 8-h fasting period (*n* = 1220); pregnant females (*n* = 79); participants without GS measurements (*n* = 304); and those with missing data for variables included in the analysis (*n* = 489). Consequently, 12,157 individuals were excluded, resulting in a final study population of 12,072 (5359 men and 6713 women) (Figure 1).

All KNHANES participants provided written informed consent to the Korea Disease Control and Prevention Agency (KDCPA). The survey procedures were approved by the Institutional Review Board of KDCPA (IRB No: 2018-01-03-P-A, 2018-01-03-C-A). Additionally, the Korea University Institutional Review Board granted exempt approval for this study, as it was classified as a secondary data analysis (IRB No: KUIRB-2025-0030-01; approved on 22 January 2025).

### 2.2. Diagnostic Criteria of Metabolic Syndrome

In this study, the diagnostic criteria for MetS were based on guidelines established by the National Cholesterol Education Program Adult Treatment Panel III [20]. However, waist circumference (WC) criteria were determined using standards proposed by the Korean Society of Obesity [21], in accordance with the International Diabetes Federation’s recommendation to apply country- and ethnicity-specific criteria. MetS was diagnosed in individuals who met three or more of the following criteria:

(1) WC ≥ 90 cm for males or ≥85 cm for females
(2) Fasting blood glucose level ≥ 100 mg/dL or the use of antidiabetic medication
(3) Blood pressure (BP) ≥ 130/85 mmHg or use of antihypertensive medications
(4) Triglyceride (TG) levels ≥ 150 mg/dL
(5) High-density lipoprotein cholesterol (HDL-C) levels < 40 mg/dL for males or <50 mg/dL for females, or the use of an antidyslipidemic medication.

### 2.3. Measurement of Relative Grip Strength

GS was measured as the force exerted by the hand while gripping an object, which involved the coordination of the four fingers and thumb. GS was assessed using a digital hand dynamometer (T.K.K.5401, TAKEI Scientific Instruments, Tokyo, Japan), and dominant hand measurements were used in this study. RGS was calculated by dividing the GS of the dominant hand by the participant’s BMI.

### 2.4. Sociodemographic and Health Behavior-Related Variables

Sociodemographic and health behavior variables were obtained from self-reported data collected from the KNHANES. The variables included in this study were sex, age, current smoking status, average alcohol consumption level, average sleep duration, physical activity level, daily caloric intake level, and frequency of resistance training per week. Physical activity level was assessed using the Global Physical Activity Questionnaire (GPAQ), which has been validated in previous population-based studies. Total physical activity level was calculated by summing the metabolic equivalent of task (MET) minutes per week across activity domains (work, transport, and leisure) [22] and categorized into low, moderate, and high levels according to the World Health Organization (WHO) guidelines. The frequency of resistance training was measured as the number of days per week that participants engaged in muscle-strengthening activities. Daily calorie intake was estimated from 24 h dietary recall data. The average sleep duration was self-reported and included as a continuous variable.

### 2.5. Anthropometric Measurements, Blood Pressure, and Biochemical Assessments

The height of the participants was measured in the standing position using a stadiometer (Seca 225, Seca, Hamburg, Germany), and their weight was measured using a digital scale (GL-6000-20, G-tech, Seoul, Republic of Korea). BMI was calculated by dividing weight (kg) by the square of height (m). WC was measured at the midpoint between the lower margin of the last rib and the superior aspect of the iliac crest, as viewed from the lateral aspect, using a measuring tape (Seca 200, Seca, Hamburg, Germany).

BP was measured using a mercury sphygmomanometer (Wall Unit 33, Baumanometer, Copiague, NY, USA), and both systolic and diastolic BP were recorded.

Blood samples were collected after a minimum of 8 h of fasting. Total cholesterol (TC), TG, low-density lipoprotein cholesterol (LDL-C), HDL-C, and fasting glucose levels were analyzed using enzymatic colorimetric methods with an automated analyzer (Hitachi Automatic Analyzer 7600-210, Hitachi Medical Corp., Tokyo, Japan). Glycated hemoglobin (HbA1c) levels were determined using high-performance liquid chromatography (HPLC) with a dedicated analyzer (Tosoh G8; Tosoh Corp., Tokyo, Japan).

### 2.6. Statistical Analysis

Categorical variables are presented as frequencies (n) and percentages (%), and continuous variables are expressed as means (M) and standard deviations (SD). To evaluate the discriminative ability of the RGS in predicting MetS, ROC curve analyses were conducted separately by sex and age group using MedCalc software (version 18.2; MedCalc Software, Ostend, Belgium). The area under the curve (AUC), sensitivity, specificity, positive predictive value (PPV), negative predictive value (NPV), accuracy, and Youden’s index were calculated for each subgroup. The optimal RGS cutoff value was determined as the point with the highest Youden index.

Based on these cutoff values, the participants were classified into two groups: an adequate relative grip strength (ARG) group and a low relative grip strength (LRG) group. Group comparisons of metabolic risk factors were conducted using independent samples *t*-tests for continuous variables and chi-square tests for categorical variables.

To further investigate the association between RGS and MetS prevalence, multivariable logistic regression analyses were conducted using two approaches: (1) RGS as a categorical variable (ARG vs. LRG) and (2) RGS as a continuous variable (per 0.1-unit increment). All models were adjusted for relevant covariates, including age, total caloric intake, group of physical activity level, current smoking status, alcohol consumption level, and frequency of resistance training. The results are expressed as odds ratios (OR) with 95% confidence intervals (CI). All statistical analyses were performed using SPSS version 26.0 (IBM Corp., Armonk, NY, USA), with statistical significance set at *p* < 0.05.

## 3. Results

### 3.1. Differences in Variables Based on the Prevalence of Metabolic Syndrome in Men and Women

The differences in various variables among participants stratified by sex and MetS status are presented in Table 1. Significant differences were observed between the non-MetS and MetS groups for both men and women across various variables.

Among men, 1733 of 5359 participants (32.34%) were diagnosed with MetS. Compared with their non-MetS counterparts, men with MetS had significantly higher values for age, weight, BMI, WC, TG, TC, LDL-C, fasting blood glucose, HbA1c, systolic blood pressure (SBP), diastolic blood pressure (DBP), average alcohol consumption level, and prevalence of MetS components (all *p* < 0.001). In contrast, men with MetS demonstrated significantly lower values for height (*p* < 0.01), HDL-C level (*p* < 0.001), RGS (*p* < 0.001), physical activity level (*p* < 0.001), and frequency of strength training per week (*p* < 0.001). No significant differences were observed in the average sleep duration or daily caloric intake between the two groups.

Among the women, 1301 of 6713 (19.38%) were diagnosed with MetS. Women with MetS had significantly higher values for age, weight, BMI, WC, TG, TC, fasting blood glucose, HbA1c, SBP, DBP, and the prevalence of MetS components than those without MetS (all *p* < 0.001). In contrast, women with MetS had significantly lower height (*p* < 0.001), HDL-C level (*p* < 0.001), RGS (*p* < 0.001), average sleep duration (*p* < 0.05), daily caloric intake (*p* < 0.05), physical activity level (*p* < 0.001), average alcohol consumption level (*p* < 0.001), and frequency of strength training per week (*p* < 0.001). Additionally, no significant differences were observed in LDL-C levels or current smoking status.

### 3.2. Results of ROC Curve Analysis of Relative Grip Strength for Predicting Metabolic Syndrome

**Figure 2:**
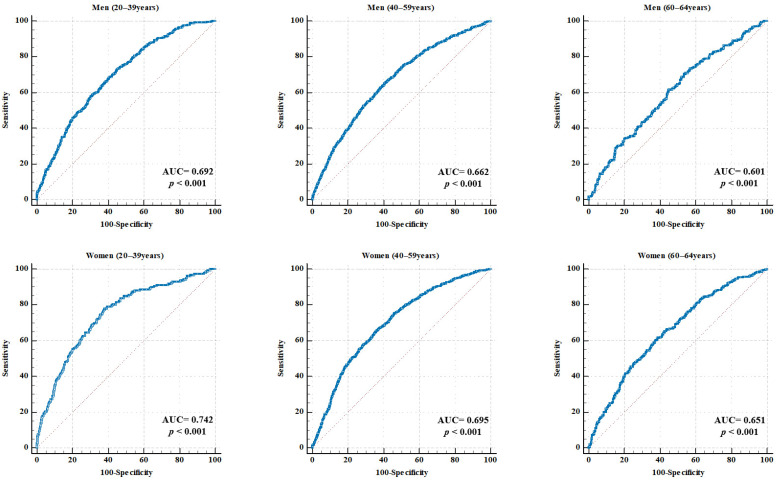
ROC curve for RGS in predicting MetS, stratified by sex and age. Each panel illustrates the ROC curves for specific subgroups of men and women aged 20–39, 40–59, and 60–64 years. The AUC and *p*-values are displayed in each graph. The red dotteed diagonal represents the line of no discrimination, indicating chance performance with an AUC equal to 0.50. AUC, area under the curve; RGS, relative grip strength; MetS, metabolic syndrome.

ROC curve analyses were conducted by sex and age group to evaluate the diagnostic utility of RGS in predicting MetS. Table 2 and Figure 2 show the AUC, optimal cutoff values, sensitivity, specificity, PPV, NPV, and overall accuracy for each subgroup.

In men, the AUC values were 0.692 (95% CI: 0.672–0.712) for the 20–39 years group, 0.662 (95% CI: 0.643–0.683) for the 40–59 years group, and 0.601 (95% CI: 0.558–0.645) for the 60–64 years group (all *p* < 0.001). The corresponding RGS cutoff values were 1.586, 1.558, and 1.520 kg/BMI. The sensitivity ranged from 65.43% to 73.09%, and the specificity ranged from 46.40% to 59.07%. The highest accuracy (61.61%) was observed in the 40–59 years group.

In women, the AUC values were 0.742 (95% CI: 0.724–0.759) for the 20–39 years group, 0.695 (95% CI: 0.680–0.710) for the 40–59 years group, and 0.651 (95% CI: 0.618–0.683) for the 60–64 years group (all *p* < 0.001). The optimal cutoff values were 0.966, 0.900, and 0.846 kg/BMI, respectively. The sensitivity ranged from 61.54% to 77.85% and the specificity ranged from 61.18% to 64.12%. The 40–59 years age group demonstrated the highest accuracy at 64.37%.

### 3.3. Differences in Variables Between Adequate and Low Relative Grip Strength Groups by Age and Sex

Participants were stratified into Adequate Relative Grip Strength (ARG) and Low Relative Grip Strength (LRG) groups based on sex- and age-specific RGS cutoff values derived from ROC analysis. Table 3, Table 4 and Table 5 summarize the differences in MetS–related variables between the groups.

Among men aged 20–39 years, the LRG group had significantly higher values of weight, BMI, WC, TG, TC, LDL-C, fasting blood glucose, HbA1c, SBP, and DBP than the ARG group (all *p* < 0.001). Conversely, height, HDL-C level, daily caloric intake, and frequency of strength training were significantly lower in the LRG than in the HGR group (all *p* < 0.001). In addition, the prevalence of all MetS components was significantly higher in the LRG than in the other groups (all *p* < 0.001).

A similar trend was observed for women aged 20–39 years. The LRG group had higher weight, BMI, WC, TG, TC, LDL-C, fasting blood glucose, HbA1c, SBP, and DBP (all *p* < 0.001). Conversely, height, HDL-C level (*p* < 0.001), and frequency of strength training were significantly lower in the LRG group (*p* < 0.01). The prevalence of MetS components was significantly higher in the LRG group (all *p* < 0.001).

Among men aged 40–59 years, significant differences were observed in nearly all metabolic indicators. The LRG exhibited higher weight, BMI, WC, TG, fasting blood glucose, HbA1c, SBP, and DBP (all *p* < 0.001) and lower HDL-C levels, frequency of strength training (*p* < 0.001), and daily caloric intake (*p* < 0.01). Additionally, the prevalence of all MetS components was significantly higher in the LRG group (all *p* < 0.001). Women in the same age group also showed significantly higher weight, BMI, WC, TG, TC, LDL-C, fasting blood glucose, HbA1c, SBP, and DBP, as well as lower height, HDL-C, physical activity level, frequency of strength training (all *p* < 0.001), and daily caloric intake (*p* < 0.05). In addition, the prevalence of all MetS components was significantly higher in the LRG than in the other groups (all *p* < 0.001).

In the 60–64 years age group, the patterns remained consistent. Both men and women in the LRG group had significantly higher weight, BMI, WC, TG, fasting blood glucose, and HbA1c levels, along with lower height and HDL-C levels (all *p* < 0.05). The prevalence of abdominal obesity, hyperglycemia, hypertriglyceridemia, hypertension, and low HDL-C levels was also significantly higher in the LRG group (*p* < 0.05).

### 3.4. Odds Ratios for Metabolic Syndrome According to Relative Grip Strength by Sex and Age Group

Logistic regression analysis was performed to examine the association between the RGS groups and the presence of MetS, using the ARG group as the reference. The analysis was stratified by sex and age. Model 1 was unadjusted, while Model 2 was adjusted for age, total caloric intake, group of physical activity level, current smoking status, frequency of strength training, and alcohol consumption level. The results are presented in Table 6.

Among men, the LRG group had significantly higher odds of MetS across all age groups. In the 20–39 years age group, the unadjusted OR was 3.300 (95% CI: 2.598–4.191), and the adjusted OR was 3.502 (95% CI: 2.658–4.614). For men aged 40–59 years, the adjusted OR was 2.738 (95% CI: 2.275–3.295), while for those aged 60–64 years, it was 1.992 (95% CI: 1.386–2.861), indicating a decreasing trend in risk magnitude with increasing age.

Among women, the LRG group demonstrated significantly higher odds of MetS across all age categories than the ARG group. For those aged 20–39 years, the unadjusted OR was 5.725 (95% CI: 3.895–8.416), and the adjusted OR was 6.846 (95% CI: 4.490–10.438), representing the strongest association among all female age groups. Women aged 40–59 years in the LRG group also showed an increased risk, with an unadjusted OR of 3.348 (95% CI: 2.831–3.961) and an adjusted OR of 2.988 (95% CI: 2.492–3.582). In the 60–64-year age group, the unadjusted OR was 2.494 (95% CI: 1.889–3.292), and the adjusted OR was 2.327 (95% CI: 1.718–3.152). All results were statistically significant (*p* < 0.001).

### 3.5. Association Between Relative Grip Strength (Continuous Variable) and the Risk of Metabolic Syndrome

To evaluate the association between RGS and the prevalence of MetS, multivariable logistic regression analyses were performed separately by sex and age group. RGS was entered as a continuous variable and scaled by a factor of 10 to facilitate interpretation; thus, the estimated OR corresponds to a 0.1-unit increase in the original RGS value. All models were adjusted for total calorie intake, physical activity level, current smoking status, alcohol consumption level, and frequency of strength training. The results are presented in Table 7, Table 8 and Table 9.

Among men, a 0.1-unit increase in RGS was associated with a 22.1% reduction in the odds of MetS in those aged 20–39 years (OR = 0.779, 95% CI: 0.745–0.814, *p* < 0.001). For men aged 40–59 years, the odds decreased by 17.1% per 0.1-unit increase in RGS (OR = 0.829, 95% CI: 0.803–0.856, *p* < 0.001), while in the 60–64-year group, the reduction was 11.4% (OR = 0.886, 95% CI: 0.833–0.942, *p* < 0.001).

In women, a stronger inverse association was observed. In the 20–39-year age group, each 0.1-unit increase in RGS was associated with 33.4% lower odds of MetS (OR = 0.666, 95% CI: 0.616–0.720, *p* < 0.001). The corresponding reductions were 25.4% in the 40–59-year group (OR = 0.746, 95% CI: 0.715–0.780, *p* < 0.001) and 22.4% in the 60–64-year group (OR = 0.776, 95% CI: 0.718–0.838, *p* < 0.001).

## 4. Discussion

This study examined the association between RGS and MetS in Korean adults using nationally representative data. Additionally, the objective was to establish sex- and age-specific cutoff values for RGS to identify individuals with an increased metabolic risk. Across all age groups and sexes, a consistent inverse association between RGS and MetS prevalence was found. Notably, the optimal cutoff values decreased progressively with age, likely reflecting physiological age-related declines in muscle strength: 1.586, 1.558, and 1.520 kg/BMI for men aged 20–39, 40–59, and 60–64 years, respectively, and 0.966, 0.900, and 0.846 kg/BMI for women in the corresponding age groups.

In the ROC analysis, the RGS demonstrated moderate discriminatory ability for predicting MetS, with AUC values ranging from 0.601 to 0.692 in men and 0.651 to 0.742 in women. These results indicate a slightly better predictive performance in women. To our knowledge, this study is the first to propose age- and sex-specific RGS cutoff values for predicting MetS in Korean adults using a nationally representative dataset. Although direct comparisons with previous studies are challenging, our results are consistent with those of previous studies conducted in the Korean population, which demonstrated a stronger association between RGS and metabolic risk in women than in men [16,23]. The decline in cutoff values with age corresponds to known sarcopenic trends, reinforcing the need for age-adjusted clinical thresholds.

Using these cutoff values, the individuals were categorized into ARG and LRG groups. The LRG had significantly worse profiles across multiple metabolic risk factors, including higher BMI, WC, TG, fasting glucose, and blood pressure, and lower HDL-C, total physical activity, and frequency of strength training. These results suggest that grip strength is closely associated not only with muscle strength but also with body composition and physical activity. In this study, participants in the LRG group reported a significantly lower frequency of strength training per week than those in the ARG group. This reduced engagement in resistance exercise may partly explain the elevated risk of MetS, as resistance training improves insulin sensitivity, regulates abdominal fat, and enhances overall metabolic profiles [24,25]. Previous studies have shown that adults with low grip strength exhibit various mechanisms linked to the pathophysiology of metabolic diseases, such as insulin resistance, chronic inflammation, intramuscular fat accumulation, mitochondrial dysfunction, and decreased myokine secretion [26,27].

Logistic regression analyses further confirmed that individuals in the LRG group had significantly higher odds of MetS than those in the ARG group, even after adjusting for key covariates. For example, women aged 20–39 years in the LRG group had 6.846 times higher odds of MetS (95% CI: 4.490–10.438), while men in the same age group had 3.502 times higher odds (95% CI: 2.658–4.614). Although the strength of the association attenuated with age, this inverse relationship remained statistically significant in nearly all subgroups. These findings align with the existing literature, showing that lower muscular strength relative to body size is a strong predictor of metabolic risk [28,29]. Previous studies using quartiles or tertiles of RGS have similarly reported a two- to five-fold increase in the odds of MetS in the lowest RGS strata [19,28,30].

Despite the practical advantages of using cutoffs for classification, cutoff-based approaches in statistical analyses remain a topic of ongoing debate. Previous studies have argued that dichotomization of continuous variables can result in information loss, potential inflation of type 1 errors, reduced statistical power, and potential misclassification [31,32]. In this context, the cutoff values proposed in the present study should be interpreted primarily as practical exploratory guidelines for screening rather than as definitive diagnostic thresholds. To address this limitation, the RGS was also analyzed as a continuous variable to preserve information and better capture dose–response patterns. In multivariable logistic regression models adjusted for various covariates, a higher RGS was independently associated with a lower risk of MetS in both sexes. For each 0.1-unit increase in the RGS, the odds of MetS decreased by 22.1% in men aged 20–39 years, 17.1% in men aged 40–59 years, and 11.4% in men aged 60–64 years. For women, the reductions were even greater at 33.4%, 25.4%, and 22.4% across the same age groups. These dose-response trends strengthen the use of RGS as a sensitive biomarker of metabolic vulnerability, corroborating the findings of cohort studies and meta-analyses [28,30].

Compared with absolute grip strength, RGS provides a more meaningful index by accounting for body size, thereby improving its predictive value for metabolic and cardiovascular outcomes [4,16,18]. Additionally, RGS is positively correlated with cardiometabolic parameters, such as blood pressure, lipid profiles, and insulin sensitivity [8].

The physiological rationale for these findings is based on the role of skeletal muscle in metabolic regulation. Skeletal muscle is the primary site for insulin-mediated glucose uptake via glucose transporter type 4 (GLUT4), and reduced muscle function contributes to insulin resistance and metabolic dysfunction [11,13]. Reduced skeletal muscle strength is associated with mitochondrial dysfunction, increased intramuscular fat accumulation, inflammation, and decreased myokine secretion, all of which contribute to insulin resistance and metabolic dysregulation [10,12,18,29]. Additionally, RGS may help identify individuals at risk of sarcopenic obesity (SO), a phenotype characterized by the coexistence of low muscle mass and high fat mass, which is associated with a higher risk of MetS than obesity alone [33,34]. Since SO is mechanistically linked to MetS through pathways such as adipose–muscle crosstalk and inflammatory signaling, RGS may serve as an integrated marker for identifying this dual-risk phenotype [33,34,35].

In this study, individuals with a low RGS reported lower levels of physical activity and strength training frequency, a behavioral pattern that is known to exacerbate metabolic profiles. These findings align with those of previous studies that have highlighted the importance of muscle fitness in maintaining metabolic health [11,33].

The strengths of this study include the use of a large, nationally representative sample of Korean adults, derivation of sex- and age-specific cutoff values, and combined application of both categorical and continuous approaches to model RGS. However, this study has several limitations. First, the cross-sectional design of this study limits its ability to draw causal inferences, underscoring the need for prospective longitudinal studies to establish temporal relationships between RGS and MetS. Second, although a population-based dataset was used, the participants were exclusively Korean, which may limit the generalizability of the findings to other ethnic or racial populations. Third, despite careful adjustment for covariates, the possibility of residual and unmeasured confounding factors remains, particularly for variables not captured in the dataset, such as stress level, sleep quality, and genetic predisposition. Fourth, selection bias may have been introduced by excluding individuals with missing grip strength or metabolic data, who may have systematically differed from those included. Fifth, self-reported data on health behaviors, such as smoking, alcohol intake, and physical activity, are subject to recall and social desirability bias, potentially resulting in misclassification. Additionally, although RGS accounts for body size by normalizing grip strength using BMI, this method may not fully reflect differences in body composition, particularly in individuals with high adiposity and low muscle mass. Finally, while the proposed cutoff values for RGS provide practical screening thresholds, they should be regarded as exploratory tools rather than definitive clinical criteria.

However, their predictive performance requires external validation in other cohorts and settings. Moreover, the AUC values derived from ROC analysis in this study ranged from 0.601 to 0.742, indicating a moderate level of predictive power. Although an AUC ≥ 0.8 is often cited as a benchmark for diagnostic excellence, such thresholds may be unrealistic for single, non-invasive physiological measures like grip strength, which are influenced by numerous biological and behavioral factors. Importantly, RGS is not intended as a stand-alone diagnostic test but as a practical screening tool in preventive settings. The robust, dose-dependent associations identified in multivariable logistic regression analyses across all sex and age groups highlight the potential value of the RGS in identifying individuals at elevated metabolic risk. Future studies may improve the predictive performance of the RGS by integrating it with other biomarkers or by developing composite indices.

## 5. Conclusions

RGS demonstrated a strong inverse association with MetS in Korean adults, with this relationship being more pronounced in women and younger age groups. While age- and sex-specific cutoff values serve as practical screening thresholds, the continuous association between RGS and metabolic risk suggests that maintaining higher muscle strength levels across the entire spectrum may benefit metabolic health. These findings highlight the importance of muscle strength assessment in evaluating metabolic risk and support the integration of resistance training into strategies for preventing metabolic diseases. The proposed RGS cutoff values should be considered as clinical screening tools rather than definitive diagnostic criteria, and their predictive utility requires validation in prospective studies.

## Figures and Tables

**Table 1:** Baseline characteristics of study participants by sex and metabolic syndrome status.

Variables	Men			Women		
	Non-MetS (n = 3626)	MetS (n = 1733)	*p*	Non-MetS (n = 5412)	MetS (n = 1301)	*p*
Age (year)	40.92 ± 13.05	48.02 ± 10.89	<0.001	42.50 ± 12.26	52.45 ± 9.84	<0.001
Height (cm)	172.69 ± 6.28	172.17 ± 6.22	0.005	159.81 ± 5.67	157.63 ± 5.79	<0.001
Weight (kg)	70.49 ± 10.49	80.27 ± 12.30	<0.001	56.85 ± 8.31	66.76 ± 11.69	<0.001
BMI (kg/m^2^)	23.60 ± 2.97	27.01 ± 3.36	<0.001	22.26 ± 3.03	26.82 ± 4.09	<0.001
WC (cm)	83.22 ± 7.96	93.58 ± 8.00	<0.001	74.99 ± 7.92	88.08 ± 9.31	<0.001
TG (mg/dL)	125.62 ± 86.51	240.08 ± 173.30	<0.001	90.31 ± 50.64	178.27 ± 109.16	<0.001
TC (mg/dL)	193.82 ± 34.45	199.91 ± 40.29	<0.001	194.86 ± 34.40	202.03 ± 42.10	<0.001
LDL-C (mg/dL)	117.88 ± 31.67	109.45 ± 40.45	<0.001	117.99 ± 30.85	119.10 ± 38.97	0.342
HDL-C (mg/dL)	50.82 ± 10.79	42.44 ± 9.84	<0.001	58.80 ± 12.06	47.29 ± 10.02	<0.001
Fasting blood glucose (mg/dL)	95.72 ± 17.43	114.95 ± 31.12	<0.001	91.85 ± 10.16	112.49 ± 28.57	<0.001
HbA1c (%)	5.48 ± 0.59	6.08 ± 1.10	<0.001	5.42 ± 0.39	6.16 ± 1.05	<0.001
SBP (mmHg)	115.42 ± 11.97	125.51 ± 14.12	<0.001	109.95 ± 13.72	125.61 ± 16.21	<0.001
DBP (mmHg)	76.78 ± 8.71	84.35 ± 10.01	<0.001	72.87 ± 8.82	80.38 ± 9.66	<0.001
RGS (kg/BMI)	1.60 ± 0.31	1.42 ± 0.30	<0.001	0.99 ± 0.23	0.82 ± 0.21	<0.001
Average Sleep duration (hours)	6.93 ± 3.94	6.84 ± 4.03	0.488	6.97 ± 2.51	6.83 ± 1.42	0.005
Daily caloric intake (kcal/day)	2445.45 ± 1020.61	2397.18 ± 986.67	0.135	1715.37 ± 694.40	1643.77 ± 659.45	0.001
Group of physical activity level [n (%)]			<0.001			<0.001
Low	1644 (45.3)	986 (56.9)		2928 (54.1)	801 (61.6)	
Moderate	1592 (43.9)	634 (36.6)		2227 (41.1)	451 (34.7)	
High	390 (10.8)	113 (6.5)		257 (4.7)	49 (3.8)	
Frequency of strength training (days/week)			<0.001			<0.001
0	2220 (61.2)	1244 (71.8)		4268 (78.9)	1135 (87.2)	
1~2	466 (12.9)	186 (10.7)		510 (9.4)	67 (5.1)	
More than 3	940 (25.9)	303 (17.5)		634 (11.7)	997 (7.6)	
Alcohol consumption level [n (%)]			<0.001			<0.001
non-drinking	955 (26.3)	380 (21.9)		2579 (47.7)	768 (59.0)	
low drinking	1584 (43.7)	643 (37.1)		2255 (41.7)	397 (30.5)	
moderate drinking	630 (17.4)	355 (20.5)		452 (8.4)	103 (7.9)	
high drinking	457 (12.6)	355 (20.5)		126 (2.3)	33 (2.5)	
Current Smoking status [n (%)]	1352 (37.3)	703 (40.7)	0.018	327 (6.0)	83 (6.4)	0.628
Components related to MetS [n (%)]						
abdominal obesity	582 (16.1)	1236 (71.5)	<0.001	514 (9.5)	857 (66.0)	<0.001
Elevated blood glucose	739 (20.4)	1322 (76.3)	<0.001	702 (13.0)	920 (70.7)	<0.001
high TG	977 (26.9)	1467 (84.7)	<0.001	752 (13.9)	1034 (79.5)	<0.001
hypertension	855 (23.6)	1316 (75.9)	<0.001	773 (14.3)	889 (68.3)	<0.001
low HDL-C	366 (10.2)	836 (48.3)	<0.001	1139 (21.3)	898 (69.3)	<0.001

Values are presented as M ± SD for continuous variables and as n (%) for categorical variables. Statistical comparisons between the non-MetS and MetS groups were performed using *t*-tests for continuous variables and chi-square tests for categorical variables. MetS, metabolic syndrome; BMI, body mass index; WC, waist circumference; TG, triglyceride; HDL-C, high-density lipoprotein cholesterol; LDL-C, low-density lipoprotein cholesterol; SBP, systolic blood pressure; DBP, diastolic blood pressure; RGS, relative grip strength; MetS, metabolic syndrome.

**Table 2:** Age- and sex-specific diagnostic performance of relative grip strength for predicting metabolic syndrome based on ROC curve analysis.

		AUC (95% CI)	Cut Off (kg/BMI)	Sensitivity (%)	Specificity (%)	PPV (%)	NPV (%)	Accuracy (%)
Men	20–39 years	0.692 (0.672–0.712) ***	1.586	73.09	54.86	27.48	89.70	58.31
	40–59 years	0.662 (0.643–0.683) ***	1.558	65.43	59.07	51.46	72.04	61.61
	60–64 years	0.601 (0.558–0.645) ***	1.520	70.43	46.40	53.27	64.40	57.56
Women	20–39 years	0.742 (0.724–0.759) ***	0.966	77.85	62.05	12.98	97.49	63.18
	40–59 years	0.695 (0.680–0.710) ***	0.900	65.27	64.12	33.65	86.88	64.37
	60–64 years	0.651 (0.618–0.683) ***	0.846	61.54	61.18	55.77	66.67	61.34

The values represent diagnostic power metrics for RGS, including AUC, optimal cutoff values, sensitivity, specificity, PPV, NPV, and overall accuracy. CI, confidence interval; kg/BMI, kilograms per body mass index unit; PPV, positive predictive value; NPV, negative predictive value. *** *p* < 0.001.

**Table 3:** Comparison of metabolic syndrome-related variables according to relative grip strength groups in individuals aged 20–39 years.

Variables	Men (20–39 Years)			Women (20–39 Years)		
	ARG [>1.586 kg/BMI] (n = 1058)	LRG [≤1.586 kg/BMI] (n = 1077)	*p*	ARG [>0.966 kg/BMI] (n = 1392)	LRG [≤0.966 kg/BMI] (n = 956)	*p*
Age (years)	30.03 ± 6.23	29.71 ± 6.04	0.231	30.39 ± 6.20	29.91 ± 6.08	0.064
Height (cm)	176.22 ± 5.61	174.28 ± 5.76	<0.001	162.75 ± 5.32	160.61 ± 5.21	<0.001
Weight (kg)	72.00 ± 10.64	80.42 ± 14.68	<0.001	55.47 ± 8.14	62.54 ± 13.09	<0.001
BMI (kg/m^2^)	23.15 ± 2.94	26.41 ± 4.21	<0.001	20.91 ± 2.65	24.20 ± 4.65	<0.001
WC (cm)	81.27 ± 8.11	89.72 ± 10.68	<0.001	71.05 ± 7.29	78.24 ± 11.13	<0.001
TG (mg/dL)	124.50 ± 90.91	152.09 ± 117.95	<0.001	81.27 ± 49.72	101.22 ± 72.61	<0.001
TC (mg/dL)	187.45 ± 33.73	194.90 ± 35.76	<0.001	182.23 ± 29.18	188.72 ± 33.06	<0.001
LDL-C (mg/dL)	111.60 ± 30.48	117.32 ± 33.00	<0.001	106.32 ± 26.08	112.40 ± 29.39	<0.001
HDL-C (mg/dL)	50.95 ± 11.08	47.13 ± 10.09	<0.001	59.66 ± 12.19	56.10 ± 11.84	<0.001
Fasting blood glucose (mg/dL)	91.99 ± 8.87	96.43 ± 19.69	<0.001	89.63 ± 10.52	92.21 ± 14.06	<0.001
HbA1c (%)	5.33 ± 0.30	5.46 ± 0.69	<0.001	5.29 ± 0.38	5.37 ± 0.56	<0.001
SBP (mmHg)	114.16 ± 10.93	117.11 ± 11.88	<0.001	105.52 ± 9.70	106.92 ± 11.60	<0.001
DBP (mmHg)	75.66 ± 9.10	78.69 ± 10.05	<0.001	70.67 ± 8.15	72.02 ± 9.39	<0.001
RGS (kg/BMI)	1.84 ± 0.19	1.32 ± 0.20	<0.001	1.17 ± 0.14	0.79 ± 0.14	<0.001
Average Sleep duration (hours)	6.96 ± 1.24	7.00 ± 5.03	0.809	7.29 ± 4.47	7.12 ± 1.38	0.236
Daily caloric intake (kcal/day)	2636.80 ± 1126.87	2369.22 ± 1037.72	<0.001	1768.92 ± 754.57	1735.87 ± 758.86	0.329
Group of physical activity level [n (%)]			0.287			0.173
Low	408 (38.6)	437 (40.6)		695 (49.9)	490 (51.3)	
Moderate	513 (48.5)	523 (48.6)		606 (43.5)	421 (44.0)	
High	137 (12.9)	117 (10.9)		91 (6.5)	45 (4.7)	
Frequency of strength training (days/week)			<0.001			<0.001
0	566 (53.5)	711 (66.0)		1068 (76.7)	793 (82.9)	
1~2	147 (13.9)	157 (14.6)		166 (11.9)	80 (8.4)	
More than 3	345 (32.6)	209 (19.4)		158 (11.4)	83 (8.7)	
Alcohol consumption level [n (%)]			0.005			0.010
non-drinking	230 (21.7)	306 (28.4)		494 (35.5)	404 (42.3)	
low drinking	541 (51.1)	513 (47.6)		694 (49.9)	421 (44.0)	
moderate drinking	134 (12.7)	119 (11.0)		152 (10.9)	95 (9.9)	
high drinking	153 (14.5)	139 (12.9)		52 (3.7)	36 (3.8)	
Current Smoking status [n (%)]	418 (39.5)	387 (35.9)	0.085	113 (8.1)	83 (8.7)	0.627
Components related to MetS [n (%)]						
abdominal obesity	158 (14.9)	501 (46.6)	<0.001	65 (4.7)	224 (23.5)	<0.001
Elevated blood glucose	164 (15.5)	249 (23.1)	<0.001	110 (7.9)	141 (14.7)	<0.001
high TG	270 (25.5)	430 (39.9)	<0.001	100 (7.2)	179 (64.2)	<0.001
hypertension	207 (19.6)	325 (30.2)	<0.001	77 (5.5)	88 (9.2)	<0.001
low HDL-C	139 (13.2)	244 (22.8)	<0.001	286 (20.8)	297 (31.6)	<0.001

Values are expressed as M ± SD for continuous variables and as n (%) for categorical variables. Statistical comparisons between the ARG and LRG groups were conducted using t-tests for continuous variables and chi-squared tests for categorical variables. ARG, adequate relative grip strength group; LRG, low relative grip strength group; BMI, body mass index; WC, waist circumference; TG, triglycerides; HDL-C, high-density lipoprotein cholesterol; LDL-C, low-density lipoprotein cholesterol; SBP, systolic blood pressure; DBP, diastolic blood pressure; MetS, metabolic syndrome.

**Table 4:** Comparison of metabolic syndrome-related variables according to relative grip strength groups in individuals aged 40–59 years.

Variables	Men (40–59 Years)			Women (40–59 Years)		
	ARG [>1.558 kg/BMI] (n = 1269)	LRG [≤1.558 kg/BMI] (n = 1307)	*p*	ARG [>0.900 kg/BMI] (n = 2026)	LRG [≤0.900 kg/BMI] (n = 1488)	*p*
Age (years)	49.14 ± 5.82	49.97 ± 5.86	<0.001	48.96 ± 5.86	50.69 ± 5.56	<0.001
Height (cm)	172.61 ± 5.29	170.05 ± 5.94	<0.001	159.80 ± 5.21	156.97 ± 5.39	<0.001
Weight (kg)	70.24 ± 9.42	75.04 ± 11.25	<0.001	56.64 ± 7.82	62.12 ± 10.38	<0.001
BMI (kg/m^2^)	23.53 ± 2.60	25.89 ± 3.23	<0.001	22.16 ± 2.70	25.18 ± 3.80	<0.001
WC (cm)	84.06 ± 7.37	90.20 ± 8.58	<0.001	75.73 ± 7.56	82.56 ± 9.48	<0.001
TG (mg/dL)	169.95 ± 152.94	195.95 ± 146.51	<0.001	104.48 ± 73.61	127.49 ± 86.93	<0.001
TC (mg/dL)	200.87 ± 35.55	201.19 ± 38.02	0.826	200.59 ± 35.08	205.44 ± 38.94	<0.001
LDL-C (mg/dL)	118.14 ± 35.89	115.84 ± 37.69	0.114	121.73 ± 32.20	126.06 ± 34.69	<0.001
HDL-C (mg/dL)	48.74 ± 11.69	46.15 ± 10.77	<0.001	57.96 ± 12.73	53.90 ± 12.30	<0.001
Fasting blood glucose (mg/dL)	103.17 ± 25.74	109.33 ± 30.02	<0.001	95.60 ± 14.98	100.62 ± 22.45	<0.001
HbA1c (%)	5.69 ± 0.82	5.95 ± 1.05	<0.001	5.56 ± 0.55	5.74 ± 0.74	<0.001
SBP (mmHg)	118.44 ± 13.84	121.15 ± 14.15	<0.001	113.45 ± 15.02	117.18 ± 16.91	<0.001
DBP (mmHg)	80.19 ± 9.40	82.22 ± 9.90	<0.001	75.15 ± 9.28	76.98 ± 9.72	<0.001
RGS (kg/BMI)	1.79 ± 0.19	1.31 ± 0.21	<0.001	1.10 ± 0.14	0.74 ± 0.13	<0.001
Average Sleep duration (hours)	6.87 ± 4.63	6.71 ± 1.18	0.238	6.79 ± 1.20	6.77 ± 1.25	0.632
Daily caloric intake (kcal/day)	2476.27 ± 951.92	2359.83 ± 992.24	0.006	1713.52 ± 637.77	1654.12 ± 677.91	0.012
Group of physical activity level [n (%)]			0.931			<0.001
Low	693 (54.6)	722 (55.2)		1102 (54.4)	907 (61.0)	
Moderate	470 (37.0)	480 (36.7)		834 (41.2)	529 (35.6)	
High	106 (8.4)	105 (8.0)		90 (4.4)	52 (3.5)	
Frequency of strength training (days/week)			<0.001			<0.001
0	810 (63.8)	954 (73.0)		1562 (77.1)	1270 (85.3)	
1~2	166 (13.1)	127 (9.7)		186 (9.2)	95 (6.4)	
More than 3	293 (23.1)	226 (17.3)		278 (13.7)	123 (8.3)	
Alcohol consumption level [n (%)]			0.881			0.004
non-drinking	301 (23.7)	328 (25.1)		1028 (50.7)	830 (55.8)	
low drinking	477 (37.6)	484 (37.0)		812 (40.1)	513 (34.5)	
moderate drinking	278 (21.9)	280 (21.4)		155 (7.7)	113 (7.6)	
high drinking	213 (16.8)	215 (16.4)		31 (1.5)	32 (2.2)	
Current Smoking status [n (%)]	533 (42.0)	517 (39.6)	0.212	107 (5.3)	69 (4.6)	0.388
Components related to MetS [n (%)]						
abdominal obesity	261 (20.6)	659 (50.6)	<0.001	230 (11.4)	533 (35.9)	<0.001
Elevated blood glucose	545 (42.9)	723 (55.3)	<0.001	494 (24.4)	520 (34.9)	<0.001
high TG	582 (41.6)	817 (62.5)	<0.001	464 (22.9)	565 (38.0)	<0.001
hypertension	529 (41.7)	712 (54.5)	<0.001	492 (24.3)	548 (36.8)	<0.001
low HDL-C	280 (22.2)	372 (28.6)	<0.001	528 (26.2)	578 (39.0)	<0.001

Values are expressed as M ± SD for continuous variables and as n (%) for categorical variables. Statistical comparisons between the ARG and LRG groups were conducted using *t*-tests for continuous variables and chi-squared tests for categorical variables. ARG, adequate relative grip strength group; LRG, low relative grip strength group; BMI, body mass index; WC, waist circumference; TG, triglycerides; HDL-C, high-density lipoprotein cholesterol; LDL-C, low-density lipoprotein cholesterol; SBP, systolic blood pressure; DBP, diastolic blood pressure; MetS, metabolic syndrome.

**Table 5:** Comparison of metabolic syndrome-related variables according to relative grip strength groups in individuals aged 60–64 years.

Variables	Men (60–64 Years)			Women (60–64 Years)		
	ARG [>1.520 kg/BMI] (n = 251)	LRG [≤1.520 kg/BMI] (n = 397)	*p*	ARG [>0.846 kg/BMI] (n = 436)	LRG [≤0.846 kg/BMI] (n = 415)	*p*
Age (years)	61.88 ± 1.37	62.04 ± 1.39	0.146	61.95 ± 1.45	61.99 ± 1.39	0.694
Height (cm)	170.09 ± 5.62	167.31 ± 5.94	<0.001	156.75 ± 5.01	154.73 ± 4.95	<0.001
Weight (kg)	67.30 ± 8.87	70.10 ± 9.96	<0.001	56.87 ± 7.57	61.55 ± 8.61	<0.001
BMI (kg/m^2^)	23.22 ± 2.51	24.99 ± 2.91	<0.001	23.12 ± 2.70	25.69 ± 3.30	<0.001
WC (cm)	84.83 ± 7.56	89.33 ± 7.95	<0.001	79.58 ± 7.32	86.16 ± 8.67	<0.001
TG (mg/dL)	150.39 ± 109.66	169.89 ± 133.83	0.044	121.85 ± 72.79	137.14 ± 77.67	0.003
TC (mg/dL)	191.51 ± 36.94	189.26 ± 38.37	0.461	203.31 ± 38.13	198.69 ± 39.37	0.084
LDL-C (mg/dL)	112.85 ± 35.44	108.20 ± 37.26	0.116	124.24 ± 35.76	119.46 ± 36.40	0.055
HDL-C (mg/dL)	48.58 ± 12.37	47.08 ± 11.69	0.120	54.70 ± 12.06	51.80 ± 12.06	0.001
Fasting blood glucose (mg/dL)	106.92 ± 20.95	112.42 ± 28.94	0.005	100.57 ± 20.71	104.53 ± 21.81	0.007
HbA1c (%)	5.85 ± 0.73	6.10 ± 1.06	<0.001	5.85 ± 0.76	6.06 ± 0.88	<0.001
SBP (mmHg)	124.52 ± 16.28	123.98 ± 14.52	0.657	122.71 ± 17.38	124.49 ± 16.24	0.124
DBP (mmHg)	78.13 ± 9.78	78.03 ± 8.39	0.886	75.75 ± 9.29	76.86 ± 9.02	0.078
RGS (kg/BMI)	1.71 ± 0.16	1.27 ± 0.21	<0.001	1.01 ± 0.13	0.67 ± 0.13	<0.001
Average Sleep duration (hours)	7.46 ± 10.17	6.81 ± 1.33	0.211	6.78 ± 1.17	6.92 ± 1.43	0.110
Daily caloric intake (kcal/day)	2311.26 ± 851.11	2225.11 ± 845.12	0.235	1654.06 ± 638.15	1555.00 ± 580.73	0.026
Group of physical activity level [n (%)]			0.113			<0.001
Low	132 (52.6)	238 (59.9)		238 (54.6)	297 (71.6)	
Moderate	100 (39.8)	140 (35.3)		178 (40.8)	110 (26.5)	
High	19 (7.6)	19 (4.8)		20 (4.6)	8 (1.9)	
Frequency of strength training (days/week)			0.010			0.012
0	152 (60.6)	271 (68.3)		348 (79.8)	362 (87.2)	
1~2	17 (6.8)	38 (9.6)		33 (7.6)	17 (4.1)	
More than 3	82 (32.7)	88 (22.2)		55 (12.6)	36 (8.7)	
Alcohol consumption level [n (%)]			0.407			0.887
non-drinking	62 (24.7)	108 (27.2)		304 (69.7)	287 (69.2)	
low drinking	92 (36.7)	120 (30.2)		109 (25.0)	103 (24.8)	
moderate drinking	63 (25.1)	111 (28.0)		20 (4.6)	20 (4.8)	
high drinking	34 (13.5)	58 (14.6)		3 (0.7)	5 (1.2)	
Current Smoking status [n (%)]	72 (29.0)	128 (32.4)	0.368	16 (3.7)	22 (5.4)	0.237
Components related to MetS [n (%)]						
abdominal obesity	57 (22.8)	182 (46.0)	<0.001	90 (20.6)	229 (55.3)	<0.001
Elevated blood glucose	134 (53.4)	246 (62.0)	0.031	167 (38.3)	190 (45.8)	0.027
high TG	114 (45.4)	231 (58.2)	0.002	222 (50.9)	256 (61.7)	0.002
hypertension	141 (56.2)	257 (64.7)	0.029	217 (49.8)	240 (57.8)	0.018
low HDL-C	53 (21.1)	114 (29.0)	0.026	155 (35.8)	193 (47.5)	0.001

Values are expressed as M ± SD for continuous variables and as n (%) for categorical variables. Statistical comparisons between the ARG and LRG groups were conducted using *t*-tests for continuous variables and chi-squared tests for categorical variables. ARG, adequate relative grip strength group; LRG, low relative grip strength group; BMI, body mass index; WC, waist circumference; TG, triglycerides; HDL-C, high-density lipoprotein cholesterol; LDL-C, low-density lipoprotein cholesterol; SBP, systolic blood pressure; DBP, diastolic blood pressure; MetS, metabolic syndrome.

**Table 6:** Logistic regression analysis of the association between relative grip strength and metabolic syndrome.

			Metabolic Syndrome		
			ARG	LRG	
				OR	95% CI
Men	20–39 years	Model 1	Reference	3.300 ***	2.598–4.191
		Model 2	Reference	3.502 ***	2.658–4.614
	40–59 years	Model 1	Reference	2.725 ***	2.313–3.209
		Model 2	Reference	2.738 ***	2.275–3.295
	60–64 years	Model 1	Reference	2.029 ***	1.467–2.808
		Model 2	Reference	1.992 ***	1.386–2.861
Women	20–39 years	Model 1	Reference	5.725 ***	3.895–8.416
		Model 2	Reference	6.846 ***	4.490–10.438
	40–59 years	Model 1	Reference	3.348 ***	2.831–3.961
		Model 2	Reference	2.988 ***	2.492–3.582
	60–64 years	Model 1	Reference	2.494 ***	1.889–3.292
		Model 2	Reference	2.327 ***	1.718–3.152

Analyses were conducted using logistic regression with ARG as the reference group. Model 1 was unadjusted. Model 2 was adjusted for age, daily calorie intake, physical activity level, current smoking status, frequency of strength training per week, and alcohol consumption level. ARG, adequate grip strength group; LRG, low grip strength group; CI, confidence interval; MetS, metabolic syndrome. *** *p* < 0.001.

**Table 7:** Multivariable logistic regression analysis of factors associated with metabolic syndrome in adults aged 20–39 years.

Variable	Men OR (95% CI)	*p*	Women OR (95% CI)	*p*
RGS	0.779 (0.745–0.814)	<0.001	0.666 (0.616–0.720)	<0.001
Age	1.122 (1.095–1.150)	<0.001	1.097 (1.060–1.135)	<0.001
Daily caloric intake	1.000 (1.000–1.000)	0.996	1.000 (1.000–1.000)	0.521
Group of physical activity level				
Low	Reference		Reference	
Moderate	0.913 (0.691–1.203)	0.520	1.028 (0.703–1.502)	0.888
High	0.899 (0.556–1.453)	0.664	1.652 (0.752–3.631)	0.211
Current smoking status	1.108 (0.840–1.460)	0.468	1.392 (0.746–2.596)	0.299
Alcohol consumption level				
Non-drinking	Reference		Reference	
Low drinking	1.020 (0.739–1.408)	0.905	1.021 (0.689–1.514)	0.916
Moderate drinking	1.278 (0.815–2.004)	0.284	1.123 (0.605–2.086)	0.713
High drinking	1.549 (1.004–2.390)	0.048	2.174 (0.909–5.198)	0.081
Frequency of strength training				
0	Reference		Reference	
1~2	0.893 (0.615–1.297)	0.552	1.247 (0.655–2.375)	0.501
More than 3	0.715 (0.495–1.032)	0.073	0.695 (0.333–1.450)	0.332

Multivariable logistic regression analysis was adjusted for age, daily caloric intake, group of physical activity level, current smoking status, alcohol consumption level, and frequency of strength training per week. RGS was entered as a continuous variable. RGS, relative grip strength; OR, odds ratio; CI, confidence interval.

**Table 8:** Multivariable logistic regression analysis of the factors associated with metabolic syndrome in adults aged 40–59 years.

Variable	Men OR (95% CI)	*p*	Women OR (95% CI)	*p*
RGS	0.829 (0.803–0.856)	<0.001	0.746 (0.715–0.780)	<0.001
Age	1.016 (1.000–1.032)	0.050	1.071 (1.054–1.089)	<0.001
Total calorie intake	1.000 (1.000–1.000)	0.753	1.000 (1.000–1.000)	0.578
Group of physical activity level				
Low	Reference		Reference	
Moderate	0.755 (0.618–0.923)	0.006	0.957 (0.791–1.159)	0.656
High	0.695 (0.485–0.994)	0.047	1.255 (0.793–1.986)	0.332
Current smoking status	1.064 (0.876–1.292)	0.532	1.772 (1.171–2.681)	0.007
Alcohol drinking level				
Non-drinking	Reference		Reference	
Low drinking	1.449 (1.137–1.846)	0.003	0.867 (0.712–1.056)	0.157
Moderate drinking	1.372 (1.041–1.807)	0.025	1.209 (.847–1.727)	0.295
High drinking	2.463 (1.824–3.327)	<0.001	1.266 (.652–2.460)	0.486
Frequency of Strength training				
0	Reference		Reference	
1~2	0.792 (0.584–1.075)	0.135	0.670 (0.457–0.981)	0.040
More than 3	0.834 (0.655–1.061)	0.139	0.712 (0.518–0.979)	0.036

Multivariable logistic regression analysis was adjusted for age, daily caloric intake, group of physical activity level, current smoking status, alcohol consumption level, and frequency of strength training per week. RGS was entered as a continuous variable. RGS, relative grip strength; OR, odds ratio; CI, confidence interval.

**Table 9:** Multivariable logistic regression analysis of factors associated with metabolic syndrome in adults aged 60–64 years.

Variable	Men OR (95% CI)	*p*	Women OR (95% CI)	*p*
RGS	0.886 (0.833–0.942)	<0.001	0.776 (0.718–0.838)	<0.001
Age	1.025 (0.906–1.160)	0.697	1.051 (0.944–1.170)	0.360
Total calorie intake	1.000 (1.000–1.000)	0.913	1.000 (1.000–1.000)	0.652
Group of physical activity level				
Low	Reference		Reference	
Moderate	0.851 (0.587–1.235)	0.396	0.932 (0.672–1.292)	0.932
High	0.230 (0.089–0.593)	0.002	0.423 (0.149–1.201)	0.106
Current smoking status	0.790 (0.540–1.156)	0.225	1.353 (0.576–3.177)	0.488
Alcohol drinking level				
Non-drinking	Reference		Reference	
Low drinking	1.650 (1.041–2.614)	0.033	0.934 (0.656–1.331)	0.707
Moderate drinking	1.786 (1.102–2.894)	0.019	1.782 (0.825–3.849)	0.141
High drinking	3.526 (1.947–6.385)	<0.001	1.873 (0.382–9.181)	0.439
Frequency of Strength training				
0	Reference		Reference	
1~2	1.414 (0.741–2.698)	0.293	0.635 (0.319–1.264)	0.196
More than 3	1.055 (0.695–1.601)	0.801	0.518 (0.301–0.0.890)	0.017

Multivariable logistic regression analysis was adjusted for age, daily caloric intake, group of physical activity level, current smoking status, alcohol consumption level, and frequency of strength training per week. RGS was entered as a continuous variable. RGS, relative grip strength; OR, odds ratio; CI, confidence interval.

## Data Availability

KNHANES data used in this study are available at https://knhanes.kdca.go.kr/knhanes/rawDataDwnld/rawDataDwnld.do# (assessed on 1 March 2025).
